# Supplementary material for: Beyond the Michaelis-Menten equation: Accurate and efficient estimation of enzyme kinetic parameters
Source: Sci Rep. 2017 Dec 5;7:17018. doi: 10.1038/s41598-017-17072-z (PMC5717222; doi:10.1038/s41598-017-17072-z)
Supplement: Supplementary file 1 — Supplementary Information [file 41598_2017_17072_MOESM1_ESM.pdf]

Beyond the Michaelis-Menten equation: Accurate and efficient estimation of enzyme kinetic parameters

Boseung Choi[1], Grzegorz A. Rempala[2], and Jae Kyoung Kim[3,*]

[1]Korea University Sejong campus, Division of Economics and Statistics, Department of National Statistics, Sejong, 30019, Korea
[2]The Ohio State University, Division of Biostatistics and Mathematical Biosciences Institute, Columbus, OH 43210, USA
[3]Korea Advanced Institute of Science and Technology, Department of Mathematical Sciences, Daejeon, 34141, Korea
[*]jaekkim@kaist.ac.kr

Supplementary Methods

Derivation of the sQ and the tQ models

The dynamics of a simple noninhibitory enzyme kinetics can be described with the following ordinary differential equations based on mass action kinetics:

$$\dot{S} = -k_f SE + k_b C, \quad \text{(S1)}$$
$$\dot{C} = k_f SE - k_b C - k_{cat} C, \quad \text{(S2)}$$
$$\dot{P} = k_{cat} C, \quad \text{(S3)}$$

where the total enzyme concentration ($E_T \equiv C + E$) and the total substrate and product concentration ($S_T \equiv S + C + P$) are conserved. One popular way to reduce the model is the standard QSSA (sQSSA) under the assumption that C rapidly equilibrates to its QSS. The QSS can be derived by solving $\dot{C} = 0$ in Eq. S2:

$$C(S) = \frac{E_T S}{K_M + S},$$

where $K_M = (k_b + k_{cat})/k_f$ is the Michaelis-Menten constant. As the QSS is determined by the states of S, by substituting the QSS to (Eqs. S1 and S3), C can be eliminated from the system and thus the following reduced model can be derived:

$$\dot{S} = -k_{cat} \frac{E_T S}{K_M + S},$$
$$\dot{P} = k_{cat} \frac{E_T S}{K_M + S}.$$

Under the validity condition of this model (Eq. 3), $S + P \approx S_T$ and thus the model can be further simplified as the following sQ model:

$$\dot{P} = k_{cat} \frac{E_T (S_T - P)}{K_M + S_T - P}.$$

Another way to reduce the model is based on the total QSSA, where the total substrate $T \equiv S + C$ instead of S is used to derive the QSS of C by solving $k_f(T - C)E - k_b C - k_{cat} C = 0$:

$$C(T) = \frac{E_T + K_M + T - \sqrt{(E_T + K_M + T)^2 - 4E_T T}}{2}.$$

Using this and the conservation $S_T = T + P$, the tQ model can be derived:

$$\dot{P} = k_{cat} \frac{E_T + K_M + S_T - P - \sqrt{(E_T + K_M + S_T - P)^2 - 4E_T(S_T - P)}}{2}.$$

Description of the Bayesian inference approach

If the complete trajectory of the product process $\{P(t)\}_{t=0}^{T}$ is observed and thus both the types and the timings of transitions are known up to a fixed time point T, the trajectory is a realization of the Markov process with exponential holding times for which the complete likelihood function can be explicitly written out[1]. Specifically, the increment of product molecules and the timing of an increment follow the Poisson process and explicitly exponential distribution[1,2] or gamma distribution with homogeneous propensity function[3], respectively. We extend this idea to derive the approximate likelihood function when available data are not complete. To this end, we consider the data $\boldsymbol{P} = (P_0, P_1, P_2, \ldots, P_m)$ over $[0, T] = [t_0, t_m]$ with the initial value $P_0 = 0$, where P_i is the scaled number of product molecules observed at time point t_i (see below for the detail about the scaling of data). During the observed time interval between t_{i-1} and t_i, an increment of molecules $n_i = P_i - P_{i-1}$ approximately follows the Poisson process with a rate parameter of $\lambda_i[t_i - t_{i-1}]$, in which λ_i is the propensity function (Tables S2 and S3). Thus the timing of the increments approximately follows the gamma distribution with the shape parameter n_i and the rate parameter λ_i. We used this idea to derive the following likelihood function for the parameters k_{cat} and K_M:

$$L(k_{cat}, K_M | \boldsymbol{P}, t_1, t_2, \ldots, t_m) \propto \prod_{i=1}^{m} \lambda_i^{n_i} [t_i - t_{i-1}]^{n_i - 1} \exp\{-\lambda_i [t_i - t_{i-1}]\}, \tag{S4}$$

where, λ_i is given by

$$\lambda_i = k_{cat} \frac{E_T(S_T - P_{i-1})}{K_M + S_T - P_{i-1}}, \text{ for the sQ model} \tag{S5}$$

or

$$\lambda_i = k_{cat} \frac{E_T + K_M + S_T - P_{i-1} - \sqrt{(E_T + K_M + S_T - P_{i-1})^2 - 4E_T(S_T - P_{i-1})}}{2}, \text{ for the tQ model.} \tag{S6}$$

Because the supports of both parameters k_{cat} and K_M are positive, for the sake of MCMC (Markov Chain Monte Carlo) inference, we assign a gamma distribution as a prior for each parameter[2]:

$$\pi(k_{cat}) \propto k_{cat}^{a_p - 1} \exp(-b_p k_{cat}), \tag{S7}$$

and

$$\pi(K_M) \propto K_M^{a_M - 1} \exp(-b_M K_M), \tag{S8}$$

where shape and rate parameters are chosen so that the prior mean is the true value and prior variance is ten times larger than the prior mean. Using these gamma priors (Eqs. S7 and S8) and the likelihood function (Eq. S4), the posterior distribution for two parameters is proportionate to

$$\pi(k_{cat}, K_M | t_1, t_2, \ldots, t_m, \boldsymbol{P}) \propto \prod_{i=1}^{m} \lambda_i^{n_i} [t_i - t_{i-1}]^{n_i - 1} \exp\{-\lambda_i [t_i - t_{i-1}]\} \times k_{cat}^{a_p - 1} \exp(-b_p k_{cat}) \times K_M^{a_M - 1} \exp(-b_M K_M).$$

The following Gibbs-sampler procedure draws samples from the above posterior distribution.

Step 1. Initialize a value of k_{cat} and K_M.

Step 2. Sample k_{cat} from the conditional posterior, gamma distribution, given the current K_M.

Step 3. Sample K_M from the conditional posterior distribution, given the current k_{cat} via the Metropolis-Hastings algorithm described below.

Step 4. Repeat steps 2–3 until convergence occurs.

To derive the conditional posterior distribution of k_{cat} given K_M, we use the fact that the propensity function λ_i (Eqs. S5 and S6) is the product of k_{cat} and a function $g_i(P_i, K_M)$, which is independent of k_{cat}:

$$\lambda_i = k_{cat} \times g_i(P_i, K_M),$$

where

$$g_i(P_i, K_M) = \frac{E_T(S_T - P_{i-1})}{K_M + S_T - P_{i-1}}, \text{ for the sQ model} \tag{S9}$$

or

$$g_i(P_i, K_M) = \frac{E_T + K_M + S_T - P_{i-1} - \sqrt{(E_T + K_M + S_T - P_{i-1})^2 - 4E_T(S_T - P_{i-1})}}{2}, \text{ for the tQ model.} \tag{S10}$$

Therefore, the conditional distribution of k_{cat} given K_M has a form of gamma distribution

$$\pi(k_{cat}|K_M, t_1, t_2, \ldots, t_m, P) \propto b_{pos}^{a_{pos}} k_{cat}^{a_{pos}-1} \exp(-b_{pos} k_{cat}), \tag{S11}$$

where $a_{pos} = a_p + \sum_{i=1}^{m} n_i$ and $b_{pos} = b_p + \sum_{i=1}^{m} g_i(P_i, K_M)[t_i - t_{i-1}]$.

On the other hand, the conditional distribution of K_M given k_{cat} has a complicated form according to

$$\pi(K_M|k_{cat}, t_1, t_2, \ldots, t_m, \boldsymbol{P}) \propto K_M^{a_M-1} \prod_{i=1}^{m} g_i(P_i, K_M)^{n_i} \exp\{-k_{cat} \sum_i g_i(P_i, K_M)[t_i - t_{i-1}] - b_M K_M\}, \tag{S12}$$

where $g_i(P_i, K_M)$ is given by Eqs. S9 and S10. To draw a sample from this conditional distribution, it is necessary to apply the intermediate Metropolis-Hastings step within the Gibbs sampler using the truncated normal distribution as the proposal distribution. The following algorithm performs the Metropolis-Hasting step at the $(l+1)$th iteration given the current (i.e. lth) K_M value.

Step 1. Draw K_M^* from the proposal distribution $j(K_M^*|K_M^{(l)}) = f(K_M^*|K_M^{(l)}, cV)$, where $f(\cdot)$ is the positive (i.e., truncated at zero) normal distribution with mean $K_M^{(l)}$ (i.e. the value of K_M at the previous iteration) and with the variance cV. Here V is the minus inverse of the second derivative of the conditional posterior (Eq. S12) at $K_M^{(l)}$ and c is a tuning constant.

Step 2. Calculate the acceptance ratio

$$A = \frac{\pi(K_M^*|k_{cat}, t_1, t_2, \ldots, t_m, \boldsymbol{P}) f(K_M^{(l)}|K_M^*, cV) F(K_M^{(l)})}{\pi(K_M^{(l)}|k_{cat}, t_1, t_2, \ldots, t_m, \boldsymbol{P}) f(K_M^*|K_M^{(l)}, cV) F(K_M^*)},$$

where $F(x)$ is cdf of $f(\cdot)$.

Step 3. Set

$$K_M^{(l+1)} = \begin{cases} K_M^* & \text{with probability min(A,1)} \\ K_M^{(l)} & \text{otherwise} \end{cases}$$

The tuning constant is set so that the acceptance ratio of the Metropolis-Hastings algorithm is about 44%, which is selected according to a criterion in[4].

MCMC sampling

We applied the proposed MCMC procedure under the Bayesian approach to the simulated timecourse data (Fig. S1). For each data set, we ran 105,000 iterations. After dropping the first 5,000 iterations for burning set, we took every 100 iterations of the remaining 100,000 iterations to obtained 1,000 approximately independent samples. As 100 datasets are used, in total we obtain the 100,000 posterior samples.

The posterior samples for the two-parameter estimation (Fig. 3) are obtained as described above. The posterior samples for the single parameter (Fig. 2) are obtained after a simple modification: when k_{cat} is sampled from conditional gamma posterior (Eq. S11), K_M is fixed as its true value and vice versa. In the MCMC procedure for joint estimation of two parameters using combined data from different experiments (Figs. 5-6), the likelihood function is modified as follows. Assume that two data sets $\boldsymbol{D}_1 = \{\boldsymbol{P}_1, t_{1,0}, t_{1,1}, \ldots, t_{1,m}\}$ and $\boldsymbol{D}_2 = \{\boldsymbol{P}_2, t_{2,0}, t_{2,1}, \ldots, t_{2,m}\}$ generated under two different conditions (e.g. low E_T and

high E_T with fixed S_T) are used together. If the likelihood functions for D_1 and D_2 as $L_1(k_{cat}, K_M|\boldsymbol{D}_1)$ and $L_2(k_{cat}, K_M|\boldsymbol{D}_2)$ respectively, then the likelihood for the combined data becomes

$$L(k_{cat}, K_M|\boldsymbol{D}_1, \boldsymbol{D}_2) = L_1(k_{cat}, K_M|\boldsymbol{D}_1) \times L_2(k_{cat}, K_M|\boldsymbol{D}_2). \tag{S13}$$

Using this modified likelihood function under the same prior gamma distributions as before, the posterior distribution is obtained by applying the MCMC method for the joint parameter estimation as described above.

Scaling of data

As discussed above, the increment of molecules of P approximately follows the Poisson process with the rate parameter being the propensity function Eqs. S5 and S6. Therefore, the likelihood function based on the timings $t_i - t_{i-1}$ approximately follows the gamma distribution with its shape parameter, which depends on the scaling of the data. Under the assumption of the Poisson process for the increment of P_i, the more accurate approximation of the likelihood is obtained when the number of molecules of the substrate is comparable to the total number of observed time points (m). Thus, throughout the work P_i are scaled so that P_m becomes the number of data sets (m) and thus E_T, S_T, and K_M are also scaled accordingly.

Sensitivity analysis and convergence checking

We performed the sensitivity analysis of the MCMC inference with respect to the prior distribution by varying prior mean and variance. The inferences are robust against changes in priors except for the lack of identifiability described in the Results section. A convergence test was performed for the joint parameter estimation from the combined data set (Fig. 7c-e). We calculated potential scale reduction factors[5] for the two parameters k_{cat} and K_M. The potential scale reduction factors are below 1.1 when the iteration number is 2,500. The Fig. S7 is the trace plot for convergence checking. We can see that the posterior sample quickly converges from five overdispersed starting points after about 300 iterations.

References

1. Choi, B. & Rempala, G. A. Inference for discretely observed stochastic kinetic networks with applications to epidemic modeling. *Biostatistics* **13**, 153–165 (2012).
2. Boys, R. J., Wilkinson, D. J. & Kirkwood, T. B. Bayesian inference for a discretely observed stochastic kinetic model. *Statistics and Computing* **18**, 125–135 (2008).
3. Wilkinson, D. J. *Stochastic modelling for systems biology* (CRC press, 2011).
4. Gelman, A., Carlin, J. B., Stern, H. S. & Rubin, D. B. *Bayesian data analysis*, vol. 2 (Chapman & Hall/CRC Boca Raton, FL, USA, 2014).
5. Gelman, A. & Rubin, D. B. Inference from iterative simulation using multiple sequences. *Statistical science* 457–472 (1992).

Reactions	Propensity functions
$S+E \xrightarrow{k_f} C$	$\frac{k_f}{\Omega} X_S X_E$
$C \xrightarrow{k_b} S+E$	$k_b X_C$
$C \xrightarrow{k_{cat}} P+E$	$k_{cat} X_C$

Table S1. Reactions and propensity functions of the original full model. $k_f = 0.017nM^{-1}s^{-1}$, $k_b = 0.03s^{-1}$, $k_{cat} = 0.0016s^{-1}$. Ω is the size of volume, and X_i is the number of molecules of species i.

Reactions	Propensity functions
$\xrightarrow{k_{cat}} P$	$\frac{k_{cat} X_{E_T} (X_{S_T} - X_P)}{X_{S_T} - X_P + K_M \Omega}$

Table S2. Reactions and propensity functions of the sQ model. $K_M = \frac{k_b + k_{cat}}{k_f}$. Ω is the size of volume, and X_i is the number of molecules of species i.

Reactions	Propensity functions
$\xrightarrow{k_{cat}} P$	$k_{cat} \frac{X_{E_T} + K_M \Omega + X_{S_T} - X_P - \sqrt{(X_{E_T} + K_M \Omega + X_{S_T} - X_P)^2 - 4X_{E_T}(X_{S_T} - X_P)}}{2}$

Table S3. Reactions and propensity functions of the tQ model. $K_M = \frac{k_b + k_{cat}}{k_f}$. Ω is the size of volume, and X_i is the number of molecules of species i.

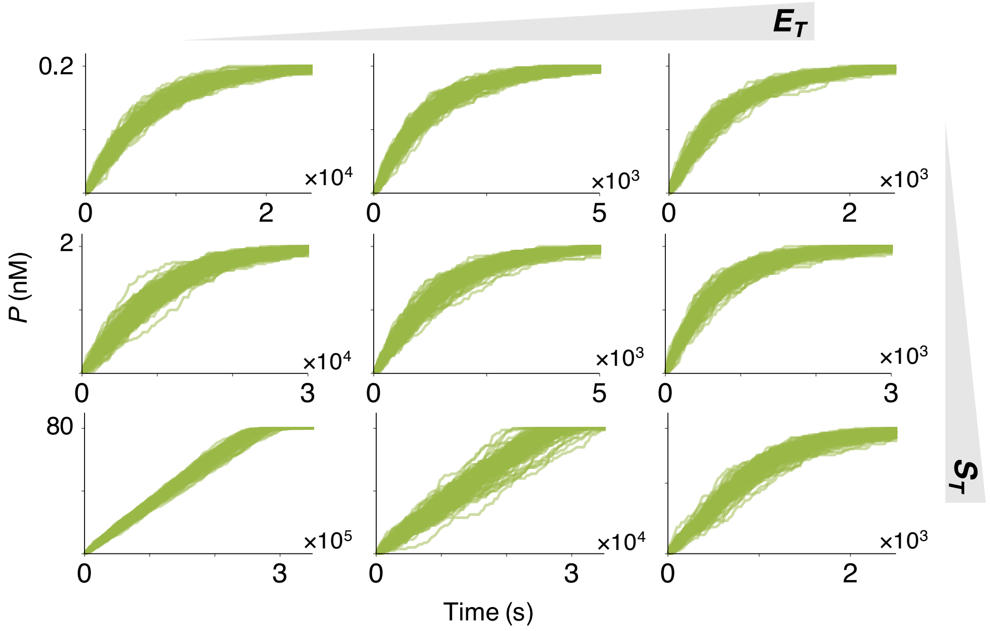

Figure S1. Simulated data sets. To obtain data sets for parameter estimation, 10^2 stochastic simulations of the original full model (Table S1) were performed for each condition: $S_T = 0.2, 2$, or $80nM$ and $E_T = 0.2, 2$, or $40nM$. Here, $S(0) = S_T$, $C(0) = 0$, and $P(0) = 0$ following a typical *in vitro* experiment protocol. Ω is chosen so that the number of initial substrate molecules becomes 80 and thus similar level of fluctuation is generated for all conditions.

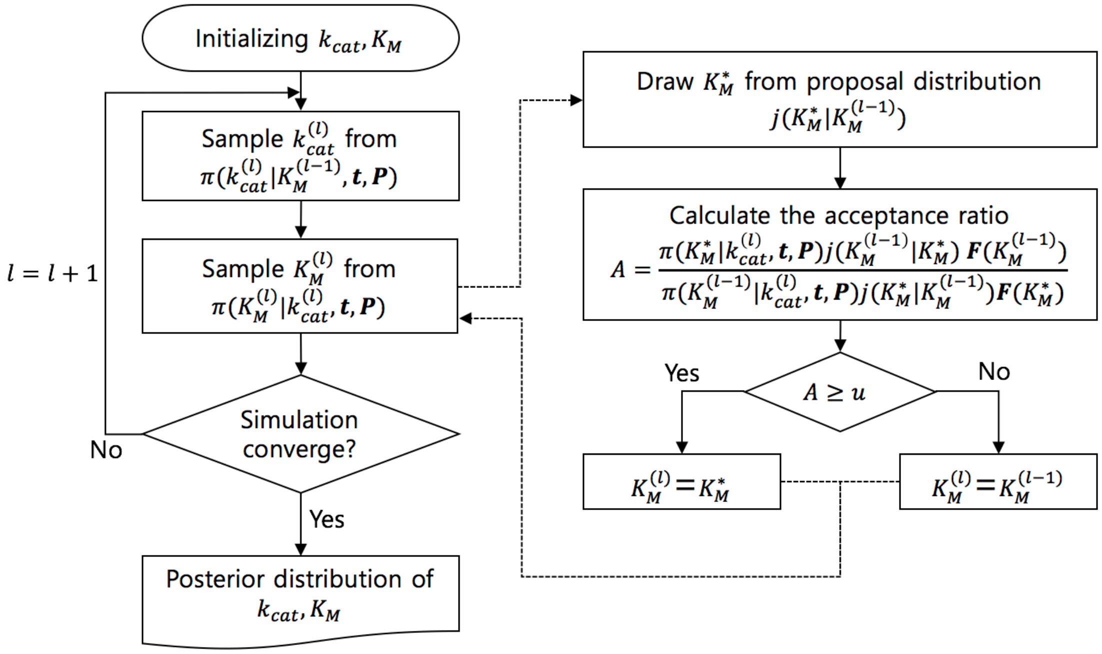

Figure S2. Schematic diagram for the Bayesian inference process estimating k_{cat} and K_M from the product progress curves. While k_{cat} can be directly sampled from the conditional gamma distribution (Eq. S11), Metropolis-Hastings algorithm is used to sample K_M. See Supplementary Methods for the details.

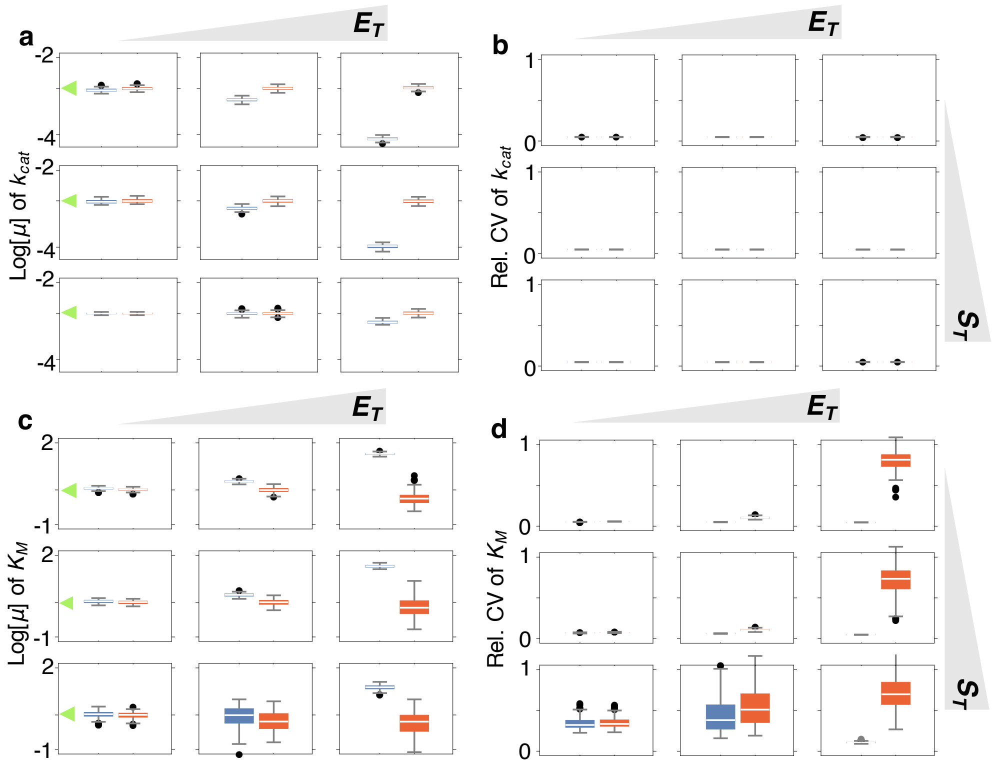

Figure S3. Box plots of Bayesian estimates of k_{cat} and K_M based on either the sQ model (left and blue) or the tQ model (right and red). (a, c) Box plots of the 10^2 posterior mean estimates of k_{cat} and K_M from 10^2 data sets (Fig. S1). Here, green triangles represent the true values of parameters. The estimates of k_{cat} and K_M obtained with the sQ model are inaccurate when E_T is large. While the estimates of k_{cat} and K_M obtained with the tQ model show little error, the confidence level of K_M estimates decreases as either E_T or S_T increases. (b, d) Box plots of 10^2 relative coefficient of variations (CVs) of the posterior distributions from 10^2 data sets (Fig. S1). Here, posterior CVs are normalized with prior CVs. When the tQ model is used, as E_T or S_T increases, so that K_M is negligible in the propensity function of the tQ model (Eq. 6), the relative CV increases, indicating the loss of identifiability.

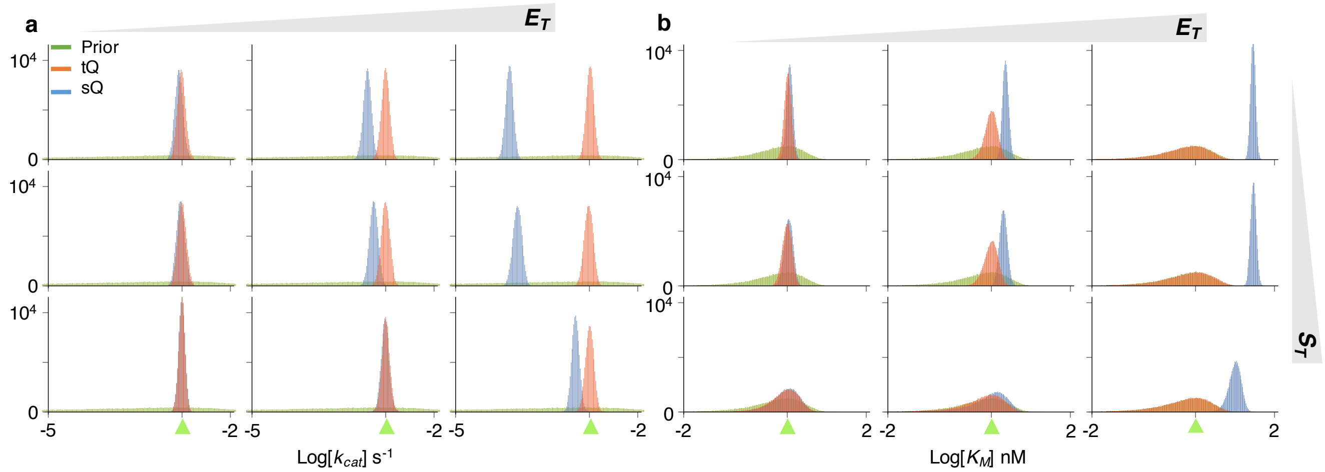

Figure S4. The estimation of a single parameter (k_{cat} or K_M) with the sQ and tQ models with an informative Gamma prior. The distributions of 10^5 posterior samples obtained with either the sQ or the tQ model for different condition ($S_T = 0.2, 2$, or 80nM and $E_T = 0.2, 2$, or 40nM). For each condition, 10^2 stochastic simulations with the full model were used as data sets (Fig. S1). Here, more informative priors are used than those in Fig. 2: gamma priors whose means are true values and variances are twice the prior mean. Despite the informative prior, estimates obtained with the sQ model still show considerable errors when E_T is large. Here, green triangles represents the true value of the parameters.

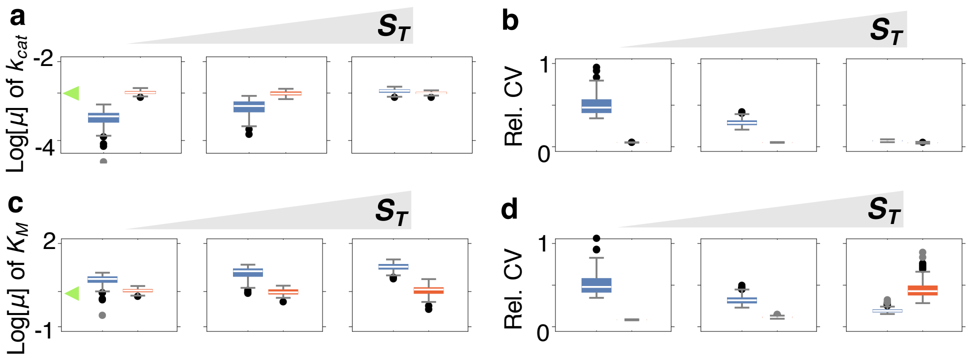

Figure S5. Box plots of Bayesian estimates of k_{cat} and K_M inferred from combined data with low and high E_T using either the sQ model (left and blue) or the tQ model (right and red) (a, c) Box plots representing the 10^2 posterior mean estimates of k_{cat} and K_M from the combined 10^2 data sets: 10^2 data sets from low E_T and 10^2 data sets from high E_T (Fig. S1). Here, $S_T = 0.2, 2$, or $80nM$. Green triangles represent the true values of parameters. The estimates of k_{cat} and K_M obtained with the sQ model have considerable errors. On the other hand, those obtained with the tQ model show little error with the confidence level similar to that of the single-parameter estimation (Figs. 2 and S3). (b, d) Box plots representing 10^2 relative CVs of the posterior distributions from the combined 10^2 data sets. Here, posterior CVs are normalized with prior CVs. When the tQ model is used, the relative CVs become similar to those of the single-parameter estimation (Fig. S3).

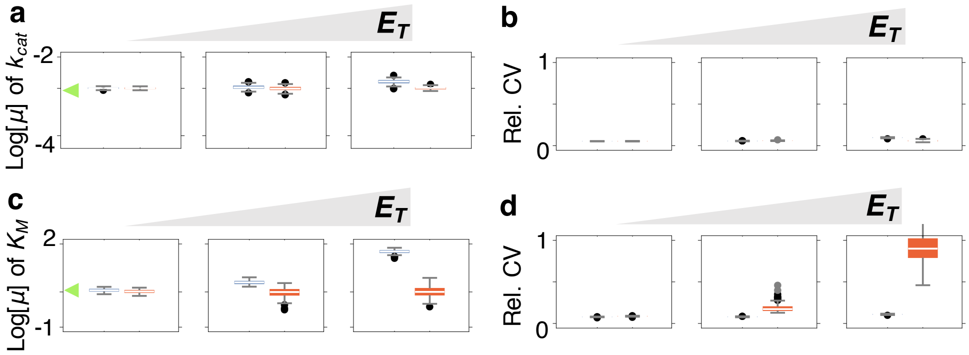

Figure S6. Box plots of Bayesian estimates of k_{cat} and K_M inferred from combined data with low and high S_T using either the sQ model (left and blue) or the tQ model (right and red) (a, c) Box plots representing the 10^2 posterior mean estimates of k_{cat} and K_M from the combined 10^2 data sets: 10^2 data sets from low S_T and 10^2 data sets from high S_T (Fig. S1). Here, $E_T = 0.2, 2$, or $40nM$. Green triangles represent the true values of parameters. The estimates of k_{cat} and K_M obtained with the sQ model have a larger error as E_T increases. On the other hand, those obtained with the tQ model show little error with the confidence level similar to that of the single-parameter estimation (Figs. 2 and S3). (B) Box plots of 10^2 relative CVs of the posterior distributions from the combined 10^2 data sets. Here, posterior CVs are normalized with prior CVs. When the tQ model is used, the relative CV becomes similar to that of the single-parameter estimation (Fig. S3).

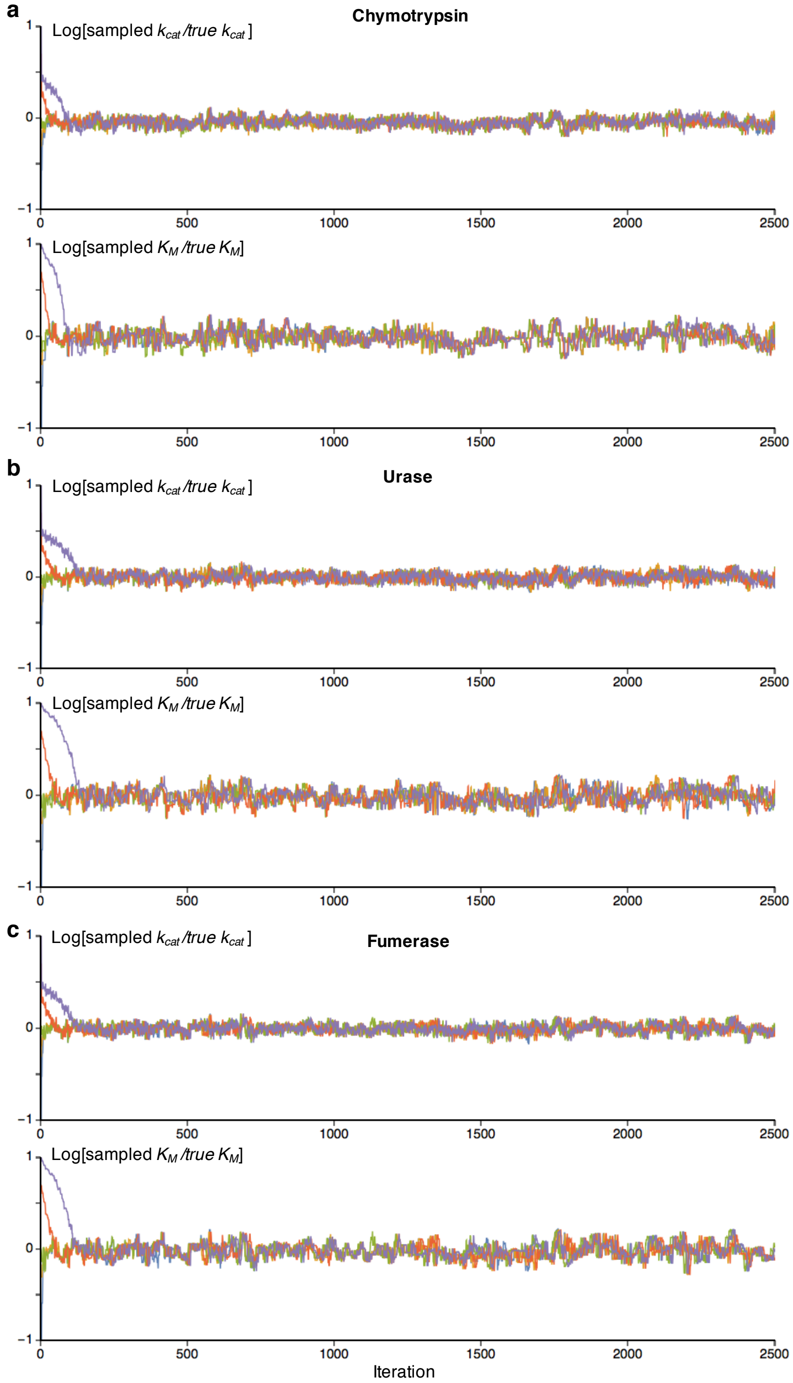

Figure S7. Trace plots of five independence posterior sample sequences of MCMC simulation used in Fig 7c. For chymotrypsin (a), urase (b), and fumarase (c), all sequences with overdispersed starting points quickly converge. Here, combined data of low E_T and high E_T is used (see Fig 7c for details).
